# Biofilm-Formation-Related Genes *csgD* and *bcsA* Promote the Vertical Transmission of *Salmonella* Enteritidis in Chicken

**DOI:** 10.3389/fvets.2020.625049

**Published:** 2021-01-14

**Authors:** Sujuan Chen, Zheng Feng, Hualu Sun, Ruonan Zhang, Tao Qin, Daxin Peng

**Affiliations:** ^1^Jiangsu Co-innovation Center for the Prevention and Control of Important Animal Infectious Disease and Zoonoses, College of Veterinary Medicine, Yangzhou University, Yangzhou, China; ^2^Joint International Research Laboratory of Agriculture and Agri-Product Safety, The Ministry of Education of China, Yangzhou, China; ^3^Jiangsu Research Center of Engineering and Technology for Prevention and Control of Poultry Disease, Yangzhou, China

**Keywords:** eggs, poultry, vertical transmission, biofilm, *Salmonella* Enteritidis

## Abstract

The contamination of *Salmonella* Enteritidis in eggs and chicken meat via vertical transmission has become a worldwide public health concern. Biofilm formation by *S*. Enteritidis further enhances its antibacterial resistance. However, whether genes related to biofilm formation affect the level of vertical transmission is still unclear. Here, *S*. Enteritidis mutants Δ*csgD*, Δ*csgA*, Δ*bcsA*, and Δ*adrA* were constructed from wild type strain C50041 (WT), and their biofilm-forming ability was determined by Crystal violet staining assay. Then the median lethal dose (LD_50_) assay was performed to determine the effects of the selected genes on virulence. The bacterial load in eggs produced by infected laying hens via the intraperitoneal pathway or crop gavage was determined for evaluation of the vertical transmission. Crystal violet staining assay revealed that *S*. Enteritidis mutants Δ*csgD*, Δ*csgA*, and Δ*bcsA*, but not Δ*adrA*, impaired biofilm formation compared with WT strain. Furthermore, the LD_50_ in SPF chickens showed that both the Δ*csgD* and Δ*bcsA* mutants were less virulent compared with WT strain. Among the intraperitoneally infected laying hens, the WT strain-infected group had the highest percentage of bacteria-positive eggs (24.7%), followed by the Δ*adrA* group (16%), Δ*csgA* group (9.9%), Δ*bcsA* group (4.5%), and Δ*csgD* group (2.1%). Similarly, among the crop gavage chickens, the WT strain group also had the highest infection percentage in eggs (10.4%), followed by the Δ*csgA* group (8.5%), Δ*adrA* group (7.5%), Δ*bcsA* group (1.9%), and Δ*csgD* group (1.0%). Our results indicate that the genes *csgD* and *bcsA* help vertical transmission of *S*. Enteritidis in chickens.

## Introduction

*Salmonella* Enteritidis is one of the most prevalent serotypes of *Salmonella* isolated from poultry and is the most commonly reported cause of human salmonellosis (1, 2). *Salmonella* can cause many acute and chronic infections in poultry, and mortality from these infections in poultry causes low yield and economic losses (3, 4). Poultry and poultry products are regarded as the main source of *S*. Enteritidis (5). Notably, chickens infected with *S*. Enteritidis frequently exhibit no clinical symptoms (6). Therefore, chicken meat and eggs from seemingly healthy animals can be contaminated by *S*. Enteritidis, which is subsequently transmitted to humans along the food cycle, resulting in a continuing public health problem (7, 8). Furthermore, vertical transmission is an essential way in which *S*. Enteritidis spreads during poultry production period (9). Previous work demonstrated that internal contamination of eggs by *S*. Enteritidis could be caused by penetration through the eggshell or by direct contamination of the egg contents before oviposition, resulting in the vertical transmission of *S*. Enteritidis from breeding chickens to commercial chickens (10). The ovaries and oviducts of the laying hens are the major *S*. Enteritidis colonization sites in which vertical transmission to eggs occurs (11).

Biofilm formation is one of the most important mechanisms utilized by *Salmonella* to survive in host cells (12). It also contributes to bacterial resistance to adverse environments and helps the bacteria to evade host immune responses (13, 14). The *in vivo* pathogenicity of *S*. Enteritidis isolated from avian sources is partially related to the biofilm formation (15). Effective biofilm formation by *S*. Enteritidis also prolongs the survival time of these bacteria and enhances their resistance to host defenses (15). The biofilm-forming ability could facilitate the spread of *S*. Enteritidis. And the consumption of poultry-derived foods is an important route of human infection by *S*. Enteritidis (16). However, it remains unclear whether the ability of *S*. Enteritidis to form effective biofilms is related to vertical transmission among laying hens. Curli fimbria (17) and cellulose (18) are important components for biofilm formation. The gene *csgD* is a central controlling regulator that can activate the transcription of *csgBAC* operons and encode the synthesis of curli fimbriae (19). This gene also promotes *adrA* gene transcription, whose product interacts with *bcsABZC*-*bcsEFG* operons to synthesize cellulose (20). Crl, an RpoS-binding factor, binding to alternative sigma factor RpoS, facilitates RNA polymerase holoenzyme formation (EσS), further to enhance CsgD expression (21). Other regulators such as MlrA or OmpR could promote CsgD expression or transcription (22, 23). Overall, the four genes *csgD, csgA, adrA*, and *bcsA* are all directly related to biofilm components and finally are selected for investigation (Table 1).

**Table 1:** Introduction of biofilm-associated genes.

**Genes**	**Function**	**References**
CsgA	Curlin major subunit, encoding sythesis of curli fimbrial which is important components of biofilm	(19)
AdrA	Diguanylate cyclase, positively regulates cellulose synthesis via production of the secondary messenger signaling molecule (3′-5′)-cyclic diguanosine monophosphate (c-di-GMP)	(24)
BcsA	Cellulose synthase, encoding sythesis of cellulose which is important components of biofilm	(25)
CsgD	Central controlling regulator, activating the transcription of *csgBAC* operons, and promote *adrA* gene transcription	(24)
RpoS	Alternative sigma factor, binding to RNA polymerase holoenzyme to facilitate CsgD expression	(17)
Crl	An RpoS-binding factor, binding to RpoS facilitates RNA polymerase holoenzyme formation (EσS)	(21)
MlrA	Positive regulator of CsgD expression	(22)
OmpR	Two-component system response regulator, facilitating *csgD* transcription	(23)

In this study, deletion mutants of *csgD, csgA, adrA*, and *bcsA* were constructed by using the λred homologous recombination method. These mutants were used to explore whether the level of *S*. Enteritidis vertical transmission in chickens is influenced by biofilm-formation-related genes.

## Materials and Methods

### Animals and Ethics Statement

Eighty one-day-old specific pathogen-free (SPF) chickens were purchased from Merial Beijing Experimental Animal Technology Co., Ltd. (Beijing, China). Ninety-six 6-month-old laying hens were purchased from Jiangsu Lihua Co., Ltd. (Changzhou, China). All *in vivo* bird experiments were performed in the negative-pressure isolators of the authorized animal biosafety level 2 (ABSL-2) facilities at Yangzhou University. All bird experiments were approved by the Jiangsu Administrative Committee for Laboratory Animals and were conducted in compliance with the guidelines of laboratory animal welfare and ethics of the Jiangsu Administrative Committee for Laboratory Animals (Permission number: SYXKSU-2016-0020).

### Bacterial Strains, Plasmids, and Growth Conditions

The strains and plasmids used in this study are listed in Table 2. Bacterial strains were routinely grown at 37°C in Luria Bertani (LB) broth or tryptic soy broth (TSB) with aeration. For strain selection, antibiotics were added at the following concentrations: 30 μg/mL chloramphenicol and 60 μg/mL ampicillin (28). Growth assays were performed in LB broth at 37°C with shaking at 220 rpm. Samples from each bacterial culture were spectrophotometrically monitored hourly for 8 h.

**Table 2:** Bacterial strains and plasmids used in this study.

**Strain or plasmid**	**Characteristics**	**References**
**Strains**		
C50041	Wild-type *Salmonella enterica*	(26)
Δ*csgD*	C50041Δ*csgD*::cat	This study
Δ*csgA*	C50041Δ*csgA*::cat	This study
Δ*adrA*	C50041Δ*adrA*::cat	This study
Δ*bcsA*	C50041Δ*bcsA*::cat	This study
DH5α	endA1hsdR17(rk-mk+) supE44 thi-1 recA1 gyrA (NalR) RelA1D(lacIZYA-argF) U169deoR (a80d lac D(lacZ) M15)	Invitrogen
**Plasmids**		
pGEM-T Easy Vector	TA Cloning Vector, Amp	Promega
pKD46	Amp, expresses k Red recombinas	(27)
pKD3	Cat gene, template plasmid	
pCP20	Expresses FLP recombinase	

### Construction of *S*. Enteritidis *csgD, csgA, adrA*, and *bcsA* Deletion Mutants

**Figure 1:**
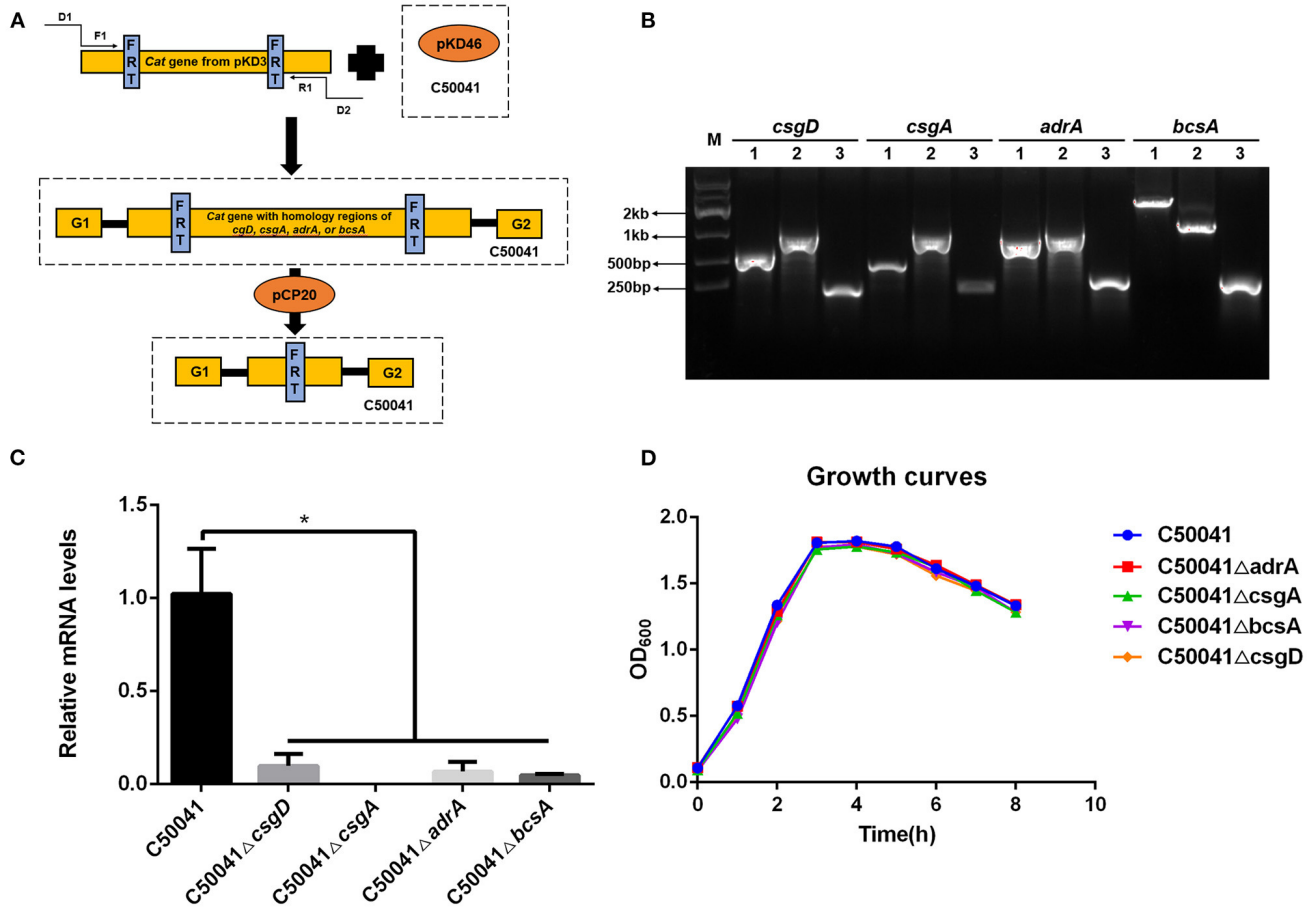
Construction of *csgD, csgA, adrA*, and *bcsA* deletion mutants. **(A)** Schematic diagram of the gene disruption strategy. D1 and D2 refer to the homology extensions or regions of *csgD, csgA, adrA*, or *bcsA*. F1 and R1 refer to the chloramphenicol resistance cassette priming sites that contain FRT sites. G1 and G2 refer to the upstream or downstream gene of the target genes. The specific steps used for constructing the mutants are described in section Construction of *S*. Enteritidis csgD, csgA, adrA, and bcsA Deletion Mutants. **(B)** PCR identification of the *S*. *enteritidis* mutant strains. (1) Wildtype strain; (2) Mutant strain containing the cat gene; (3) Mutant strain without the cat gene; M, marker. **(C)** Transcriptional levels of *csgD, csgA, adrA*, and *bcsA* genes. Means and standard deviations from three independent experiments are shown; *p < 0.05. **(D)** Growth curves of *S*. *enteritidis* WT and mutant strains (C50041Δ*csgD*, C50041Δ*csgA*, C50041Δ*bcsA*, and C50041Δ*adrA*).

Deletions of *csgD, csgA, adrA*, or *bcsA* from the chromosome of *S*. Enteritidis C50041 were performed by using gene replacement methods based on the λRed recombinase system (29); the primers used in these protocols are listed in Table 3. The *S*. Enteritidis mutant strains C50041Δ*csgD*, C50041Δ*csgA*, C50041Δ*adrA*, and C50041Δ*bcsA* were constructed as shown in Figure 1A. The four genes *csgD, csgA, adrA*, and *bcsA* were amplified by PCR. The pKD3-encoded chloramphenicol resistance cassette was amplified using the primers csgD-D1/csgD-D2, csgA-D1/csgA-D2, adrA-D1/adrA-D2, and bcsA-D1/bcsA-D2 (Table 3). C50041 harboring plasmid pKD46 was electroporated with the csgD cat, csgA cat, adrA cat, or bcsA cat amplicon with 0.2% (wt/vol) L-arabinose at 30°C. Chloramphenicol-resistant transformants were selected at 37°C and were confirmed to have lost pKD46 on the basis of sensitivity to ampicillin conferred by the auxiliary plasmid pCP20. The successful creation of the mutants C50041Δ*csgD*, C50041Δ*csgA*, C50041Δ*adrA*, and C50041Δ*bcsA* was subsequently confirmed via PCR using primers in Table 3, respectively, followed by sequencing of the PCR products for verification.

**Table 3:** Primers designed and used in this study.

**Primers**	**Prime sequence (5' → 3')**	**Purpose**
csgD-F	CATGTTTAATGAAGTCCATAGTAGTC	Amplify gene *csgD*
csgD-R	TTACCGCCTGAGATTATCGTTTG	
csgA-F	TTACCATGAAACTTTTAAAAGTGGC	Amplify gene *csgA*
csgA-R	TTAATACTGGTTAGCCGTGGCGTTGTT	
adrA-F	ACCGAAAAGCGGTTGAACAG	Amplify gene *adrA*
adrA-R	GGTTACGTCCGGCATTCTTT	
bcsA-F	ATGAGCGCCCTTTCCCGGT	Amplify gene *bcsA*
bcsA-R	TCATTGTTGAGCCTGAGCCAT	
csgD-D1	TCCATAGTAGTCATGGTCACACACTATTGTTGATCACAAAGCCATCTCTGgtgtaggctggagctgcttc	Amplify *cat* cassette of gene *csgD*
csgD-D2	CGCCTGAGATTATCGTTTGCCCATGAAACTGCCTGGGTGCGATTTTTGACcatatgaatatcctccttag	
csgA-D1	ATGAAACTTTTAAAAGTGGCAGCATTCGCAGCAATCGTAGTTTCTGGCAGgtgtaggctggagctgcttc	Amplify *cat* cassette of gene *csgA*
csgA-D2	GCCAAAACCAACCTGACGCACCATTACGCTGGAATCAGATGCGGTCTGATcatatgaatatcctccttag	
adrA-D1	TGTTAGTGTCGCAACCTGTCTTTGGCGGCTGGTGGCTATTGCTGGTCGGCgtgtaggctggagctgcttc	Amplify *cat* cassette of gene *adrA*
adrA-D2	ACTTCGGTGCGGTTACGTCCGGCATTCTTTGCTTTGTAAAGCGCCATATCcatatgaatatcctccttag	
bcsA-D1	TGAGCATCCGCGCTGGCAGCGTATTCGCGACGAGCATAAAGCACTTTATCgtgtaggctggagctgcttc	Amplify *cat* cassette of gene *bcsA*
bcsA-D2	CATTGTTGAGCCTGAGCCATAACCCGATCCGACGGCTGTATCGCCGCTTGcatatgaatatcctccttag	
QgyrB-F	ACGCGTCTGTTGACCTTCTTC	Quantitative real-time PCR
QgyrB-R	CTGTTCCTGCTTACCTTTCTTCAC	
QcsgD-F	CGGCCGGTTGCATTGTTTTA	
QcsgD-R	CCACGTGTTCCTGGTCTTCA	
QcsgA-F	TCGACCAGTGGAACGCTAAAA	
QcsgA-R	ACCAACCTGACGCACCATTAC	
QadrA-F	GGCCATTAAATTAGCGGAAC	
QadrA-R	AATAAAATTTCCCAGTGGCG	
QbcsA-F	CGGGCGTGAATCATTTCGTC	
QbcsA-R	TCAGGAACCAGCCCATTGTC	

### Quantitative Real-Time PCR (qRT-PCR) Analysis

Bacteria were grown in 1/10 TSB medium at 28°C for 24 h in 60 mm dishes (Corning). The supernatant was discarded and the bacteria accumulated in the biofilms under the dishes were scraped. The total RNA was extracted using a Bacterial RNA Kit (Omega). The cDNA was synthesized using a PrimeScript RT reagent Kit with gDNA Eraser (Takara) and quantified via TB Green Premix Ex Taq (Takara). The gene transcript levels were tested in triplicate for real-time PCR in a Linegene 9600 Plus machine (Bioer). Primer pairs of Qgyrb-F/R, QcsgD-F/R, QcsgA-F/R, QadrA-F/R, and QbcsA-F/R (Table 3) were used for the mRNA detection of *gyrB, csgD, csgA, adrA*, and *bcsA*, respectively. The target genes' mRNA levels were normalized to the *gyrB* mRNA levels (2^−ΔΔCt^) (30).

### Detection of Biofilm Formation

Biofilm formation by *S*. Enteritidis was detected by using the crystal violet staining quantitative method (28). For biofilm formation, a single bacterial colony was inoculated into 3 mL of TSB culture medium and subjected to shaking cultivation at 220 rpm at 37°C overnight, then this culture fluid was diluted 1/100 with 1/10 TSB diluent and then transferred to a 96-well U-shaped cell culture plate (100 μL/well) for static cultivation at 28°C for 24 h. For detection, the biofilm was stained with crystal violet, a 3:1 alcohol-acetone solution was used to dissolve the crystals, and the optical density at 550 nm (OD_550_ value) was measured on a microplate reader (Tecan, Switzerland).

### Challenge of SPF Chickens With Wild Type Strain or Mutant *S*. Enteritidis Strains

WT strain and its mutants were inoculated on a solid LB plate and statically cultivated at 37°C for 16–18 h. A few rich bacterial colonies were processed by the streak plate method and statically cultivated at 37°C for 3 h. Phosphate-buffered saline (PBS) containing 15% glycerol was used to wash the bacterial lawn. Eighty one-day old chickens were randomly divided into 16 groups (*n* = 5/group), with three groups per bacterial strain and one negative control group. Chickens from the challenged groups were intraperitoneally injected with 0.1 mL of bacterial fluid containing differing amounts of colony-forming units (CFU, Table 5). Chickens from the negative control group were intraperitoneally injected with 0.1 mL of PBS containing 15% glycerol. After 2 weeks of continual observation, the median lethal dose (LD_50_) was calculated by applying a modified Karber's method (31).

### Experiments to Test *S*. Enteritidis Vertical Transmission Among Laying Hens

#### The Grouping of Laying Hens

Laying hens (96 birds, 6-month-old) in a peak period of egg production were randomly divided into six groups, each of which included a crop gavage subgroup (*n* = 8/subgroup, 10^9^ CFU/chicken) and an intraperitoneal injection subgroup (*n* = 8/subgroup, 2 × 10^7^ CFU/chicken). The negative control group was injected with PBS containing 15% glycerol. To determine whether the hens were previously exposed to *Salmonella*, blood samples were collected before challenge, and the resulting sera were tested with a diagnostic antigen (Diagnostic antigen for *salmonella*, China Veterinary Drug Supervision Institute, China) for *Salmonella*.

#### Detection of Eggs Contaminated by Wild Type Strain or Mutant *S*. Enteritidis Strains

The egg production quantity was recorded for 9 days before the injection. Two weeks after bacterial injection, the egg production was quantified for 9 days. For evaluating the level of vertical transmission, the *S*. Enteritidis load was detected in eggs produced every day (2 weeks). After sterilizing eggshells with 75% ethyl alcohol, the contents of the eggs were collected and sealed in sterile bags, and then incubated at 37°C for 48 h. The egg contents were subsequently inoculated onto MacConkey agar plates for 24 h at 37°C. The resulting suspected bacterial colonies were verified by using diagnostic serum (Diagnostic sera for *salmonella*, Sanshui Inspection Equipment Co. LTD, China) for *Salmonella*. This detection was performed continuously over 2 weeks.

### Statistics

The results were analyzed with GraphPad Prism 8 software (San Diego, CA, USA) and are expressed as the means ± s.d. An unpaired Student's two-sided *t*-test was employed to determine the differences between the two groups. Differences with a *p* < 0.05 were considered to be significant. A chi-square test was applied to analyze the percentages of eggs in which bacteria were positively detected.

## Results

### Identification of Generated *S*. Enteritidis Mutants

The successful generation of *S*. Enteritidis mutant strains C50041Δ*csgD*, C50041Δ*csgA*, C50041Δ*adrA*, and C50041Δ*bcsA* was confirmed by using PCR (Figure 1B). After each target gene had been replaced by the chloramphenicol resistance gene, the size of the altered chromosome was estimated to be about 1.2 kb. After the resistance gene had been knocked out, the size of the gene was observed to be about 200–400 bp, which is consistent with the expected value (Table 4). The transcriptional levels of *csgD, csgA, adrA*, and *bcsA* genes were determined by relative qRT-PCR. The results showed that the four genes' transcriptional levels were significantly reduced in the mutants compared with the WT strain (Figure 1C). The growth rates of the *S*. Enteritidis mutant strains were similar to that of the wild type (WT) strain (Figure 1D).

**Table 4:** Sizes of PCR-amplified fragments.

**Name of** **gene**	**Wild** **strain (1)**	**Mutant strain** **containing cat** **gene (2)**	**Mutant strain** **without cat** **gene (3)**
*csgD*	652 bp	1,132 bp	202 bp
*csgA*	461 bp	1,146 bp	216 bp
*adrA*	1,059 bp	1,217 bp	272 bp
*bcsA*	2,628 bp	1,307 bp	308 bp

*Number 1, 2, and 3 are listed in Figure 1B, representing different lanes*.

### Biofilm Formation of the *S*. Enteritidis Mutants

**Figure 2:**
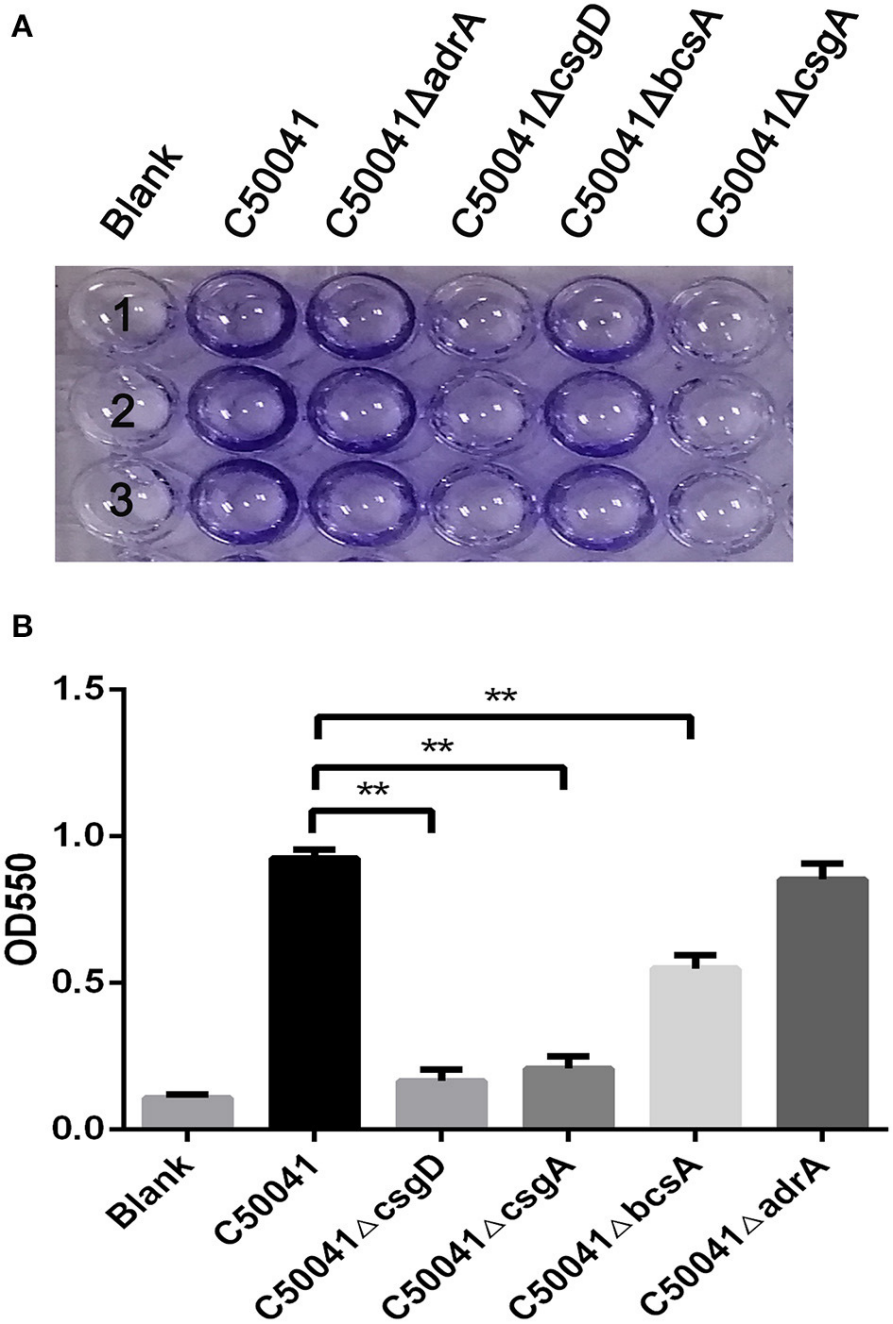
Measurement of *S*. *enteritidis* biofilms. **(A)** Crystal violet staining of bacteria grown on 96-well-plates. Overnight cultures were diluted in 1/10 TSB, and 100 μL of each bacterial suspension was added to a 96-well-plate, then incubated at 28°C for 24 h. Results were observed after staining with 0.4% crystal violet. **(B)** Quantification of the crystal violet staining by optical density (OD_550_) measurement. Means and standard deviations from three independent experiments are shown; **p < 0.01. Number 1, 2, and 3 means repetition of different strains in the same detection.

To examine the effects of the deleted genes on wild type strain biofilm formation, crystal violet staining tests were conducted. The WT strain C50041 and its mutant strains C50041Δ*adrA* and C50041Δ*bcsA* had similar amounts of circular staining in the plate well walls, whereas C50041Δ*csgD* and C50041Δ*csgA* had almost no circular staining (Figure 2A).

Quantifying the crystal violet staining results revealed that the OD_550_ values of C50041Δ*csgD*, C50041Δ*csgA*, and C50041Δ*bcsA*, but not that of C50041Δ*adrA*, were significantly lower than the OD_550_ value of the wild type strain (all *p* < 0.01), suggesting that biofilm formation was blocked after single mutations of *csgD, csgA*, or *bcsA* (Figure 2B).

### Bacterial Virulence in SPF Chickens

#### Mortality Rate/LD_50_

As shown in Table 5, the group challenged with a dose of 1.67 × 10^6^ CFU of the wild type strain had 60% mortality. In contrast, none of the SPF chickens died after challenge with C50041Δ*csgD* at any of the tested infection doses. Furthermore, the LD_50_ value of the wild type strain was 1.33 × 10^6^ CFU, whereas that of C50041Δ*csgD* was >1.50 × 10^6^ CFU, suggesting that the ability of *S*. Enteritidis to form a biofilm increased its bacterial virulence. Notably, the LD_50_ value of C50041Δ*bcsA* (1.42 × 10^6^ CFU) and C50041Δ*adrA* (>1.70 × 10^6^), but not that of C50041Δ*csgA* (9.69 × 10^5^ CFU), was also higher than the LD_50_ value of the wild type strain.

**Table 5:** LD_50_ of the wide type and gene-deletion mutant *S*. *enteritidis* strains in SPF chickens.

**Strain**	**Group No**.	**Challenge dose (CFU)**	**Qty of animals**	**Death count**	**Death rate**	**LD_**50**_ (CFU)**
	1	1.67 × 10^6^	5	3	60%	
C50041	2	1.67 × 10^5^	5	0	0%	1.33 × 10^6^
	3	1.67 × 10^4^	5	0	0%	
	1	1.50 × 10^6^	5	0	0%	
Δ*csgD*	2	1.50 × 10^5^	5	0	0%	>1.50 × 10^6^
	3	1.50 × 10^4^	5	0	0%	
	1	1.22 × 10^6^	5	3	60%	
Δ*csgA*	2	1.22 × 10^5^	5	0	0%	9.69 × 10^5^
	3	1.22 × 10^4^	5	0	0%	
	1	1.70 × 10^6^	5	1	20%	
Δ*adrA*	2	1.70 × 10^5^	5	1	20%	>1.70 × 10^6^
	3	1.70 × 10^4^	5	0	0%	
	1	1.79 × 10^6^	5	2	40%	
Δ*bcsA*	2	1.79 × 10^5^	5	0	0%	1.42 × 10^6^
	3	1.79 × 10^4^	5	1	20%	

### Detection of *S*. Enteritidis Vertical Transmission

#### Egg Production From the Infected Laying Hens

**Figure 3:**
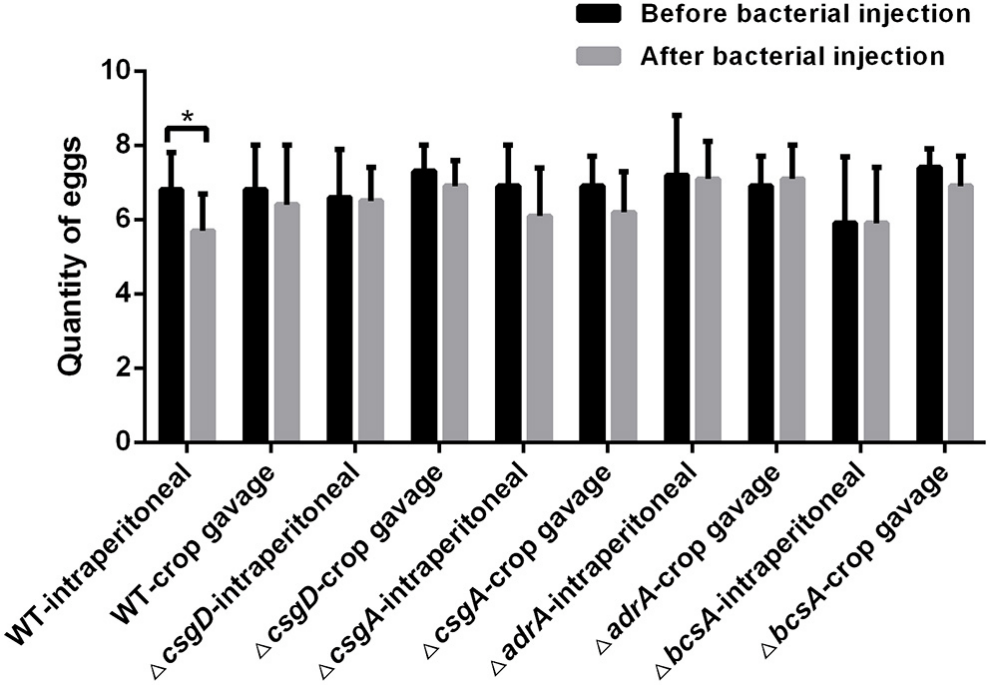
Quantity comparison of egg production by the laying hens before and after injection of wild type strain and deletion mutants. Laying hens (96 birds, 6-month-old) in a peak period of egg production were randomly divided into six groups, each of which included a crop gavage subgroup (*n* = 8/subgroup, 10^9^ CFU/chicken) and an intraperitoneal injection subgroup (*n* = 8/subgroup, 2 × 10^7^ CFU/chicken). The negative control group was injected with PBS containing 15% glycerol. The egg production quantity was recorded for 9 days before and after the bacterial injection. Finally, the average of egg production was compared (*p < 0.05).

Before the laying hens were injected with bacteria, none of their serum samples agglutinated with diagnostic *Salmonella* antigens, suggesting these hens did not have a *Salmonella* infection. Two weeks after bacterial injection, the egg production was quantified for 9 days. As shown in Figure 3, the egg production capacity of the group infected with C50041 intraperitoneally (6.8 ± 1.0) was significantly lower than that of the non-infected group (*p* < 0.05). In contrast, there was no significant difference between the egg production of the non-infected group and the groups infected with any of the other mutants (*p* > 0.05).

#### Bacterial Load in Eggs Produced by the Infected Laying Hens

After bacterial challenge, the bacterial load count in eggs produced by the infected laying hens was assessed daily. As shown in Table 6, among the intraperitoneally infected animals, the wild type-infected group had the highest infection percentage in eggs (24.7%), followed by the C50041Δ*adrA* group (16%), C50041Δ*csgA* group (9.9%), C50041Δ*bcsA* group (4.5%), and C50041Δ*csgD* group (2.1%). Similarly, among the crop gavage chickens, the wild type group also had the highest infection percentage in eggs (10.4%), followed by the C50041Δ*csgA* group (8.5%), C50041Δ*adrA* group (7.5%), C50041Δ*bcsA* group (1.9%), and C50041Δ*csgD* group (1.0%). Interestingly, the percentages of produced eggs were higher in the intraperitoneal injection subgroup than in the crop gavage subgroup. Together, these data indicate that the genes *csgD* and *bcsA* are closely related to the level of *S*. Enteritidis vertical transmission from infected laying hens to eggs.

**Table 6:** Bacterial detection in eggs from the infected laying hens.

**Num. of** ** days after** ** bacterial** ** injection**	**C50041**				**Δ*****csgD***				**Δ*****csgA***				**Δ*****adrA***				**Δ*****bcsA***			
	**Intraperitoneal** ** injection**		**Crop gavage** ** injection**		**Intraperitoneal** ** injection**		**Crop gavage** ** injection**		**Intraperitoneal** ** injection**		**Crop gavage** ** injection**		**Intraperitoneal** ** injection**		**Crop gavage** ** injection**		**Intraperitoneal** ** injection**		**Crop gavage** ** injection**	
	**Qty of** ** eggs with** ** bacteria/** **total** ** eggs**	**Positive** ** rate**	**Qty of** ** eggs with** ** bacteria/** **total** ** eggs**	**Positive** ** rate**	**Qty of** ** eggs with** ** bacteria/** **total** ** eggs**	**Positive** ** rate**	**Qty of** ** eggs with** ** bacteria/** **total** ** eggs**	**Positive** ** rate**	**Qty of** ** eggs with** ** bacteria/** **total** ** eggs**	**Positive** ** rate**	**Qty of** ** eggs with** ** bacteria/** **total** ** eggs**	**Positive** ** rate**	**Qty of** ** eggs with** ** bacteria/** **total** ** eggs**	**Positive** ** rate**	**Qty of** ** eggs with** ** bacteria/** **total** ** eggs**	**Positive** ** rate**	**Qty of** ** eggs with** ** bacteria/** **total** ** eggs**	**Positive** ** rate**	**Qty of** ** eggs with** ** bacteria/** **total** ** eggs**	**Positive** ** rate**
1	0/5	0	0/5	0	0/6	0	0/7	0	1/7	14.29%	0/7	0	0/7	0	0/8	0	0/6	0	1/8	12.50%
2	2/4	50%	5/7	71.43%	2/6	33.33%	0/6	0	2/5	40%	0/7	0	1/8	12.50%	0/7	0	1/8	12.50%	0/7	0
3	1/6	16.67%	1/5	20%	0/6	0	0/8	0	0/8	0	0/6	0	0/6	0	2/6	33.33%	1/4	25%	0/7	0
4	1/6	16.67%	0/7	0	0/7	0	0/6	0	0/7	0	0/5	0	3/8	37.50%	0/6	0	0/5	0	0/7	0
5	4/4	100%	0/7	0	0/6	0	0/7	0	0/3	0	0/7	0	1/5	20%	0/8	0	0/7	0	0/7	0
6	0/5	0	4/5	80%	0/8	0	0/7	0	1/6	16.67%	2/7	28.57%	2/8	25%	3/8	37.50%	0/3	0	1/7	14.29%
7	0/4	0	0/2	0	0/6	0	0/7	0	0/4	0	0/3	0	1/6	16.67%	0/6	0	0/5	0	0/5	0
8	2/6	33.33%	0/6	0	0/5	0	0/6	0	1/6	16.67%	2/6	33.33%	2/8	25%	0/7	0	1/6	16.67%	0/8	0
9	4/7	57.14%	0/8	0	0/7	0	0/7	0	1/7	14.29%	0/7	0	2/8	25%	0/7	0	0/8	0	0/7	0
10	2/7	28.57%	0/7	0	0/7	0	0/6	0	0/6	0	0/6	0	0/6	0	0/6	0	0/6	0	0/8	0
11	1/7	14.29%	0/8	0	0/8	0	0/7	0	1/6	16.67%	2/7	28.57%	1/8	12.50%	1/6	16.67%	0/5	0	0/7	0
12	1/6	16.67%	0/6	0	0/5	0	1/8	12.50%	0/6	0	0/7	0	2/7	28.57%	0/7	0	0/7	0	0/6	0
13	0/6	0	0/8	0	0/7	0	0/8	0	0/7	0	0/7	0	1/6	16.67%	1/8	12.50%	1/8	12.50%	0/7	0
14	2/6	33.33%	0/8	0	0/7	0	0/7	0	0/7	0	1/6	16.67%	1/7	14.29%	0/8	0	0/6	0	0/7	0
15	1/6	16.67%	0/7	0	0/6	0	0/7	0	1/6	16.67%	1/6	16.67%	0/8	0	1/8	12.25%	0/5	0	0/6	0
Total	21/85	24.7%^a^	10/96	10.4%^a^	2/97	2.1%^d^	1/104	1.0%^c^	9/91	9.9%^bc^	8/94	8.5%a	17/106	16%^ab^	8/106	7.5%^ab^	4/89	4.5%^cd^	2/104	1.9%^bc^

*Values marked with different letters are significantly different (p < 0.05)*.

## Discussion

Bacteria within biofilms can enhance the resistance to adverse environments and prolonged survival. Cellulose and curli fimbria are both very important for biofilm formation by *Salmonella* bacteria (32). A review by Simm et al. suggests that most chronic infections are associated with the biofilm formation of microorganisms (19). In a *S*. Typhimurium model, the immune response to curli is site specific, and oral administration of curli ameliorates the damaged intestinal epithelial barrier and reduces the severity of colitis (33). Mauricio et al. found that preventing cellulose synthesis increased *S*. Typhimurium virulence, whereas stimulation of cellulose synthesis inside macrophages decreased the virulence (34). So far, whether biofilm-associated genes regulate the virulence in *S*. Enteritidis is unclear. Therefore, in this study, the *S*. Enteritidis genes *bcsA* and *csgA*, which encode cellulose and curli fimbria, respectively (19), as well as the genes *csgD* and *adrA*, which regulate the biofilm-related genes (20), were selected for investigation. We report here that mutants of *S*. Enteritidis with deletion in *csgD, csgA*, and *bcsA*, but not of *adrA*, display defects in the level of biofilm formation by *S*. Enteritidis. Our results also suggest that genes related to biofilm formation (*csgD, csgA, bcsA*, and *adrA*) can alter the virulence of *S*. Enteritidis differently. The mortality rates of chickens infected with these strains indicate that deletions of *csgD* or *bcsA* attenuated the virulence of WT strain whereas a deletion of *csgA* yielded the opposite result. Although the deletion of *adrA* had limited effects on the biofilm formation of *S*. Enteritidis, the LD_50_ value of the C50041Δ*adrA* mutant was much higher than that of the WT strain. These data reveal that biofilm formation is related to bacterial virulence, and in *S*. Enteritidis, synthesis of curli and cellulose could enhance its virulence.

*S*. Enteritidis can be transmitted vertically through laying hens (35) and may cause persistent infection. Although previous studies have shown that the biofilms may be related to persistent *Salmonella* infections (36), the relationship between biofilms and the vertical transmission of *S*. Enteritidis is still unclear. Here, we deleted four genes known to be related to biofilm formation and studied their roles in the vertical transmission of *S*. Enteritidis among laying hens. The results of our vertical transmission assay indicate that the genes *csgD* and *bcsA* significantly enhance the level of *S*. Enteritidis vertical transmission, whereas the genes *csgA* and *adrA* have limited effects. Within groups infected with the same *S*. Enteritidis strain, the percentages of produced eggs were higher in the intraperitoneal injection subgroup than in the crop gavage subgroup, which is consistent with the trends reported previously (37). Considering the biofilm makes the bacteria stuck somewhere, we speculated that biofilm could help the *Salmonella* better and longer survival in the reproductive tract or on the egg or associated environment. In adverse, deletion of cellulose encoded by *bcsA* prevented biofilm formation, further decreased the adaption of *S*. Enteritidis in produced eggs, resulting in a decrease in bacterial penetration through the eggshell or by direct contamination of the egg contents before oviposition.

As the central regulator of biofilm formation, *csgD* regulates the expression of CsgA and AdrA, AdrA further controls the BcsA expression (17). Therefore, deletion of *csgD* could affect the biofilm formation. However, *bcsA*, in addition to being controlled by *adrA*, also can be regulated by other regulators, including the second messenger c-di-GMP and sigma factor RpoS (19). Therefore, deletion of *adrA* had limited effects on biofilm formation. Interestingly, cellulose, encoded by *bcsA*, might be more important for the vertical transmission of *S*. Enteritidis compared with *csgA*-encoded curli fimbria, another biofilm component. These data are in line with our above virulence results and imply a different biological function between these two components. Overall, of the four genes studied here, *csgD* and *bcsA* had the strongest impacts on *S*. Enteritidis vertical transmission, the potential mechanisms will be studied in future.

In conclusion, we studied the impacts of four *S*. Enteritidis biofilm-associated genes on the vertical transmission in chickens by constructing gene-deletion mutants. Our results indicate that biofilm-associated genes *csgD* and *bcsA* may play important roles in the vertical transmission of *S*. Enteritidis. These findings lay the foundation for a better understanding how to control the vertical transmission of *S*. Enteritidis.

## Data Availability Statement

The original contributions presented in the study are included in the article/supplementary materials, further inquiries can be directed to the corresponding author/s.

## Ethics Statement

The animal study was reviewed and approved by Jiangsu Administrative Committee for Laboratory Animals.

## Author Contributions

SC, HS, and DP conceived research. HS and RZ performed research. SC, ZF, HS, and DP analyzed data. SC, ZF, HS, and TQ wrote the paper. All authors contributed to the article and approved the submitted version.

## Conflict of Interest

The authors declare that the research was conducted in the absence of any commercial or financial relationships that could be construed as a potential conflict of interest.
